# Changes of the Freshwater Microbial Community Structure and Assembly Processes during Different Sample Storage Conditions

**DOI:** 10.3390/microorganisms10061176

**Published:** 2022-06-08

**Authors:** Yunfeng Wang, Xinghao Li, Yong Chi, Weibo Song, Qingyun Yan, Jie Huang

**Affiliations:** 1 Institute of Evolution & Marine Biodiversity, College of Fisheries, Ocean University of China, Qingdao 266003, China; yunfengwang1@126.com (Y.W.); yongchi94@foxmail.com (Y.C.); wsong@ouc.edu.cn (W.S.); 2 Donghu Experimental Station of Lake Ecosystems, Key Laboratory of Aquatic Biodiversity and Conservation of Chinese Academy of Sciences, Institute of Hydrobiology, Chinese Academy of Sciences, Wuhan 430072, China; 3 Key Laboratory of Regional Development and Environmental Response, Hubei Engineering Research Center for Rural Drinking Water Security, Hubei University, Wuhan 430062, China; li_xhao@163.com; 4 Laboratory for Marine Biology and Biotechnology, Qingdao National Laboratory for Marine Science and Technology, Qingdao 266237, China; 5 Environmental Microbiomics Research Center, School of Environmental Science and Engineering, Southern Marine Science and Engineering Guangdong Laboratory (Zhuhai), State Key Laboratory for Biocontrol, Sun Yat-sen University, Guangzhou 510006, China; yanqy6@mail.sysu.edu.cn

**Keywords:** prokaryotic microbial communities, eukaryotic microbial communities, freshwater, storage conditions, community assembly

## Abstract

A long-standing dilemma for microbial analyses is how to handle and store samples, as it is widely assumed that the microbial diversity and community patterns would be affected by sample storage conditions. However, it is quite challenging to maintain consistency in field sampling, especially for water sample collection and storage. To obtain a comprehensive understanding of how sample storage conditions impact microbial community analyses and the magnitude of the potential storage effects, freshwater samples were collected and stored in bottles with lid closed and without lid at room temperature for up to 6 days. We revealed the dynamics of prokaryotic and eukaryotic microbial communities under different storage conditions over time. The eukaryotic microbial communities changed at a faster rate than the prokaryotic microbial communities during storage. The alpha diversity of the eukaryotic microbial communities was not substantially influenced by container status or storage time for up to 12 h, but the beta diversity differed significantly between the control and all treatment samples. By contrast, no significant changes of either the alpha or beta diversity of the prokaryotic microbial communities were observed within 12 h of room-temperature storage, regardless of the container status. The potential interactions between microbial taxa were more complex when samples were stored in sealed bottles, and the deterministic processes played an increasingly important role in shaping the freshwater microbial communities with storage time. Our results suggest that water samples collected and stored without refrigeration for no more than 12 h may still be useful for downstream analyses of prokaryotic microbial communities. If the eukaryotic microbial communities are desired, storage of water samples should be limited to 3 h at room temperature.

## 1. Introduction

Microorganisms are ubiquitous on earth and play crucial roles in the biogeochemical cycles of different ecosystems [1,2,3]. Advances in DNA sequencing technologies and bioinformatics have revolutionized the study of the diversity and dynamics of environmental microbial communities [4,5,6]. However, biases can be introduced at almost all stages (i.e., sampling, sample storage and processing, DNA extraction and sequencing) of the experimental process and affect the perception and interpretation of the microbial community [7,8,9,10]. Maintaining consistency in each processing step is therefore essential during a study. A long-standing dilemma for microbial analyses is how to handle and store samples, as microorganisms are highly sensitive and can respond rapidly to the environmental changes [11,12,13]. Thus, it is reasonable to assume that the microbial diversity and community patterns would be affected by sample storage conditions.

Although there are evidences showing that room-temperature storage even for a few days would not strongly affect the overall bacterial community structure of soil or fecal samples [14,15], the low temperature storage guidelines have been widely accepted and followed to minimize the impact of storage conditions on the microbial communities. For example, keeping samples frozen or using a preservative are considered the best alternative solutions for sample storage during shipping, when it is not feasible to immediately process samples after sampling [16,17,18]. But these methods cannot be uniformly applied to large volumes of water samples, and thus water samples have to be stored at room temperature for varying duration time in some cases (i.e., remote fieldwork). So far, however, how and whether storage of water samples at room temperature impacts microbial community analyses requires a comprehensive investigation.

In addition, water samples are routinely stored in bottles before subsequent processes, but the storage conditions varied among cases and studies, such as the volume ratio of enclosed water and the resulting headspace, with the lid closed or not. It is widely known that microbial respiration will substantially reduce the dissolved oxygen, which may in turn change the community structures [19,20], and thus keeping a large headspace in the bottles or leaving the lids of sampling bottles open to ensure sufficient oxygen has become a common practice in field sampling. However, detailed changes in microbial diversity, structure and community assembly processes in response to these potential storage effects on time scales of hours to days remain unclear. It should also be pointed out that the impacts of bottle containment on microbial prokaryotic assemblages have long been recognized [21]. Significant changes in community composition, species abundance, and metabolic activity of marine bacteria were frequently observed during bottle storage [22]. The effects of bottle confinement on microbial eukaryotic assemblages can also be found. The community structure of marine protist was dramatically changed in response to bottle containment during a 3-day period [12,13]. In terms of the biomass, the rapid shifts from autotrophic to heterotrophic marine picoplankton detected in the bottles may result from biased estimates and do not take place in natural conditions [11,23]. The increasing evidence suggests that bottle incubations may induce variations in microbial community structure that do not reflect the initial community [13]. Nevertheless, very few studies have taken both prokaryotic and eukaryotic microorganisms as a whole community into consideration, especially in freshwater habitats.

In this study, freshwater samples were collected and stored in bottles with lid closed and without lid at room temperature from 3 h to 6 days. The prokaryotic and eukaryotic microbial diversity was then analyzed by high-throughput sequencing of 16S and 18S rRNA genes. Specific aims were (i) to clarify how the freshwater microbial communities change during storage in sampling bottles with lid closed and without lid at room temperature and explore the dynamics of potential interactions between microbial taxa over time and (ii) to investigate the ecological processes driving microbial community assembly under different storage conditions.

## 2. Materials and Methods

### 2.1. Experimental Design and Sampling

Water samples were collected from the second largest urban lake of China, Lake Donghu (30°30′07″ N, 114°21′45″ E), which was located less than 200 m from our laboratory. Within 15 min, a total of 60 L lake water were sampled, transported to the laboratory, and thoroughly mixed. The water samples were aliquoted immediately (1 L per aliquot) and stored in plastic bottles (1 L) with lid closed (CO) and without lid (UC) at room temperature without direct sunlight for 3, 6, 12, 24, 48, 96 and 144 h. Each treatment, set up in triplicate, was serially filtered through 20 μm pore-size nylon filters (Millipore, Ireland) and through 0.22 μm pore-size PVDF membranes (Millipore, Ireland). Another 3 samples were filtered immediately after being aliquoted as control replicates (CN). All filters were immediately stored at −80 °C until DNA extraction.

### 2.2. DNA Extraction, PCR Amplification and Sequencing

Total genomic DNA was extracted using the DNeasy PowerWater kit (Qiagen, Beverly, MA, USA), according to the manufacturer’s instructions. The hypervariable V4 region of the 16S and 18S rRNA genes was amplified using the primers ArBa515F (5′-GTGCCAGCMGCCGCGGTAA-3′) and 806R (5′-GGACTACHVGGGTWTCTAAT-3′), and EK-565F (5′-GCAGTTAAAAAGCTCGTAGT-3′) and EK-1134R (5′-TTTAAGTTTCAGCCTTGCG-3′), respectively [24,25,26]. Triplicate PCR reactions were performed in 20 μL volumes containing 0.2 μL of BSA, 0.4 μL of FastPfu Polymerase (TransGen AP221-02), 0.8 μL of each primer (5 μM), 2 μL of dNTPs (2.5 mM), 4 μL of 5× FastPfu buffer (2.5 units/μL) and 10 ng of template DNA. Cycling parameters of the 18S amplicon included an initial denaturation at 95 °C for 3 min, followed by 35 cycles of 30 s at 95 °C, 30 s at 55 °C, 45 s at 72 °C and a final extension of 10 min at 72 °C. The same PCR program was used to obtain the 16S amplicon, with the change of 27 cycles being set. PCR products were visualized by 1% agarose gels and purified using the AxyPrep DNA Gel Extraction Kit (Axygen Biosciences, Union City, CA, USA). Purified PCR products were pooled and sequenced using 2 × 300 bp paired-end sequencing approach on the Illumina MiSeq platform (Majorbio, Shanghai, China), according to the manufacturer’s protocols.

### 2.3. Sequence Data Processing and Taxonomic Assignment

Paired-end reads were assembled with FLASH [27] and trimmed with Trimmomatic [28], and then the sequences were quality-filtered using QIIME v1.8 [29]. Chimeric sequences were identified and removed using the Chimera UCHIME algorithm [30] before downstream analysis. Quality-checked sequences were classified into operational taxonomic units (OTUs) using UPARSE [31] with the 97% sequence similarity cutoff. OTU reference sequences were blasted against the Silva 16S rRNA database (http://www.arb-silva.de (accessed on 1 December 2019)) and PR^2^ 18S rRNA database [32]. Singletons and metazoan OTUs were excluded prior to further analysis. Samples were rarefied to even sequencing depth by random resampling prior to subsequent statistical analyses [33].

### 2.4. Network Analysis

Network analysis was performed to investigate the interactions among microbial taxa using the phylogenetic Molecular Ecological Network Analysis (MENA) pipeline (http://ieg4.rccc.ou.edu/mena (accessed on 15 January 2021)) based on the Random Matrix Theory (RMT) [34]. Only the OTUs occurring in more than 50% samples were retained for subsequent analysis, and the networks were constructed with an identical similarity threshold (0.92). A positive correlation may indicate a mutualistic interaction, whereas a negative correlation may imply predation or competition among the taxa [35,36]. The co-occurrence networks were visualized using Gephi (0.9.2, Mathieu Bastian & Sebastien Heymann, Paris, France) [37]. The connectivity of each node was confirmed by its within-module connectivity (*Zi*) and among-module connectivity (*Pi*) [38,39]. The potential keystone OTUs were identified by *Zi* and *Pi* [34], and one-way analysis of variance (ANOVA) was used to determine differences in the relative abundance of potential keystone taxa between groups.

### 2.5. Neutral Community Model

To evaluate the potential importance of stochastic processes on microbial community assembly, the neutral community model (NCM) was applied to predict the relationship between the occurrence frequency of OTUs and their relative abundance across the wider metacommunity [40]. In this model, the OTUs were separated into three partitions according to their occurrence frequency, i.e., more frequent (above partition), less frequent (below partition) and within the 95% confidence interval (neutral partition), in which the calculation of the 95% confidence interval is based on 1000 bootstrap replicates. The parameter R^2^ quantifies the overall fit level to the NCM, and m describes immigration rate. When R^2^ approaches 1, it indicates that the community assembly is completely determined by the stochastic processes, while R^2^ ≤ 0 indicates the community assembly does not fit to the NCM [41]. All computations for the NCM were performed in R (version 3.6.3).

### 2.6. Community Co-Occurrence Pattern

The checkerboard score (C-score) was calculated to explore the dominant co-occurrence patterns during succession, given that the matrix is relatively unaffected by small changes in data [42,43]. Firstly, the sequence table was converted into a binary matrix of presence (1) and absence (0), and then an SIM9 randomization algorithm was used for C-score calculation based on 30,000 simulations with a burn-in of 500 iterations and sequential swap randomization algorithm from the R package “EcoSimR” in R (version 3.6.3) [44,45,46]. The standardized effect sizes (SES) for C-score were evaluated by the difference between the observed index and the mean of the simulated index divided by the standard deviation of the simulated index [47]. A positive SES value represents separation, whereas a negative SES value suggests an overall aggregated pattern [48]. Furthermore, insignificant SES values will be between −2 and 2, significantly greater than expected SES values will be >2 and significantly less than expected SES values will be <−2 [47,49].

### 2.7. Statistical Analyses

Statistical analyses were implemented using the R-Studio interface to R (version 3.6.3), and visualized by the “ggplot2” package [50]. Alpha diversity indices, including OTU richness and Shannon diversity index, were calculated based on the identified OTUs using the “vegan” package, and the differences in alpha diversity indices were evaluated using one-way analysis of variance (ANOVA) [51,52]. Significant differences of the relative abundances at different taxonomic levels were performed by Welch’s *t*-test based on STAMP [53]. Non-metric multidimensional scaling (NMDS) ordination was performed based on the Bray–Curtis distances to investigate the overall dissimilarity of microbial communities. Analysis of similarities (ANOSIM), permutational multivariate analysis of variance (PERMANOVA) and multiple-response permutation procedure (MRPP) based on Bray–Curtis distances were conducted to test the differences of microbial communities between storage conditions. Mantel tests were performed to reveal the Spearman’s rank correlation between the Bray–Curtis dissimilarity of microbial communities and the storage time based on Euclidean distances. Principal component analysis (PCA) was carried out using the “prcomp” function in R package “ggbiplot”.

## 3. Results

### 3.1. Effect of Sample Storage on Community Composition

A total of 1,922,491 and 1,612,295 high-quality prokaryotic and eukaryotic sequences were obtained from 45 samples, which clustered into 3553 and 905 OTUs at the 97% similarity level, respectively. After filtering, each sample was rarified to the same sequencing depth (*n* = 27,744 for 16S; *n* = 22,420 for 18S). Good’s coverage values ranged from 0.983 to 0.998 for each sample, indicating that the majority of the microbial taxa had been recovered.

**Figure 1:**
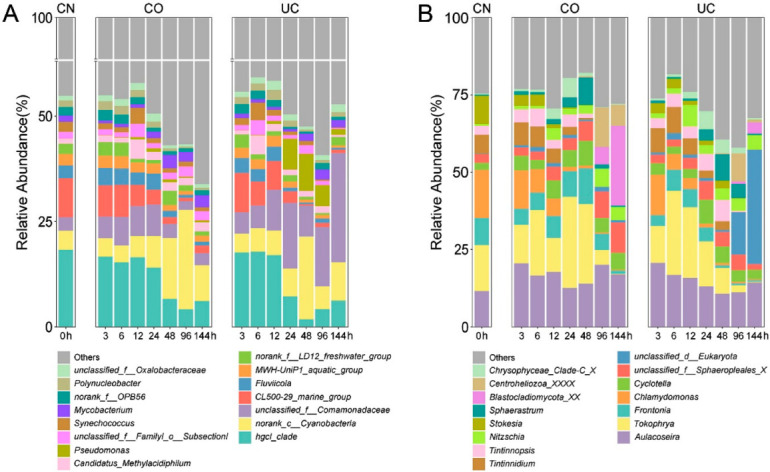
Stacked bar charts of the relative abundance of the prokaryotes (**A**) and eukaryotes (**B**) at the genus level. Shown are the averages from *n* = 3. CN: control group, CO: with lid closed, UC: without lid.

In the control group (CN), Actinobacteria was the most abundant phylum (in terms of sequencing reads), accounting for 37.13% (±0.40%) of the total bacteria abundance, followed by Proteobacteria (27.47 ± 4.28%), Bacteroidetes (14.73 ± 1.45%) and Cyanobacteria (10.71 ± 5.56%) (Figure S1A). The relative abundances of Actinobacteria, Bacteroidetes, Chlorobi and Firmicutes decreased with the increase of storage time, whereas those of Proteobacteria, Cyanobacteria, Verrucomicrobia, Chloroflexi and Planctomycetes increased (Figure S1A). At the genus level, the relative abundance of *Pseudomonas*, *Mycobacterium* and norank_c_Cyanobacteria increased with storage time, while the opposite trend was observed for the genera hgcI_clade, CL500-29_marine_group, *Fluviicola*, MWH-UniP1_aquatic_group and *Polynucleobacter* (Figure 1A).

**Figure 2:**
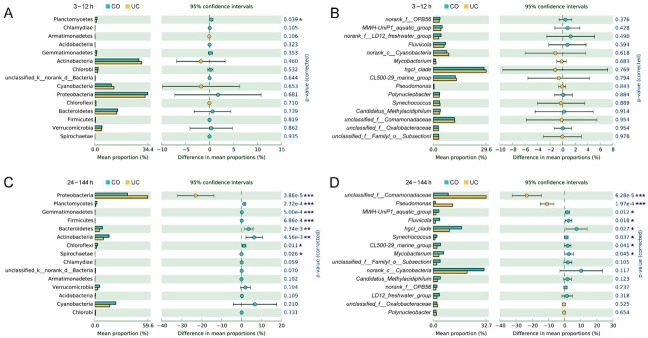
Significant differences of relative abundances of the top 15 prokaryotic phyla (**A**,**C**) and genera (**B**,**D**) between CO (with lid closed) and UC (without lid) groups. Shown are the averages from *n* = 3. *: 0.01 < *p* ≤ 0.05, **: 0.001 < *p* ≤ 0.01, ***: *p* ≤ 0.001.

During the first 12 h, the relative abundances of top 15 prokaryotic taxa (at phylum and genus levels) were not significantly different between the two storage conditions, except for the phylum Planctomycetes (Figure 2A,B). During 24–144 h, the proportion of Proteobacteria (*Pseudomonas* and unclassified Comamonadaceae) was statistically lower in the group with lid closed (CO) compared to the samples without a lid (UC), whereas Actinobacteria (hgcI_clade, CL500-29_marine_group and *Mycobacterium*), Bacteroidetes (*Fluviicola*), Chloroflexi, Planctomycetes, Gemmatimonadetes, Firmicutes and Spirochaetae exhibited the opposite trend (*p* < 0.05, Figure 2C,D). Additionally, the relative abundances of *Synechococcus* and MWH-UniP1_aquatic_group were also significantly higher in the CO group (*p* < 0.05, Figure 2D).

Ciliophora, Chlorophyta and Ochrophyta were the most prevalent eukaryotic phyla in the control group, accounting for 57.15 ± 3.44%, 20.37 ± 5.93% and 16.51 ± 4.37% of the total reads, respectively (Figure S1B). The relative abundances of Ciliophora (*Tokophrya*, *Frontonia*, *Tintinnidium* and *Stokesia*) and Chlorophyta (*Chlamydomonas*) declined with increasing storage time, while the proportions of Ochrophyta (*Cyclotella* and *Nitzschia*) and Centroheliozoa (*Sphaerastrum*) increased over time (Figure 1B and Figure S1B).

During the first 12 h, the relative abundances of all top 15 eukaryotic taxa were not statistically different between the CO and UC groups, except for the phylum Stramenopiles_X and two genera, *Nitzschia* and Chrysophyceae_Clade-C_X. (*p* < 0.05, Figure S2A,B). After samples were stored for 24 h, the relative abundances of the phyla Ciliophora, Stramenopiles_X, Mesomycetozoa, and unclassified_k_Alveolata and the genera *Stokesia* and unclassified_d_Eukaryota were significantly lower in samples with lid closed (CO) than in the UC group; however, the opposite trend was observed for Fungi, Cercozoa, Centroheliozoa, Lobosa, Streptophyta, unclassified_k_Opisthokonta and *Frontonia* (*p* < 0.05, Figure S2C,D).

### 3.2. Effect of Sample Storage on Microbial Diversity

**Figure 3:**
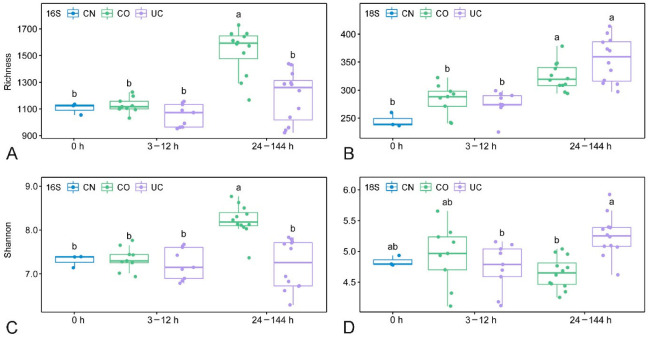
The boxplots showing richness and Shannon index of prokaryotes (**A**,**C**) and eukaryotes (**B**,**D**) under different storage conditions. Different letters indicate a statistical significance (*p* < 0.05, ANOVA) between different groups. CN: control group, CO: with lid closed, UC: without lid.

No significant changes in richness and Shannon diversity were observed for either prokaryotes or eukaryotes during storage for up to 12 h, regardless of the storage conditions (Figure 3). With increasing storage time (24–144 h), the alpha diversity indices of prokaryotes were significantly higher in the group with lid (CO) than in the UC group (*p* < 0.05, Figure 3A,C), whereas the opposite pattern was observed in the eukaryotic microorganisms, as indicated by the Shannon index (*p* < 0.05, Figure 3D).

**Figure 4:**
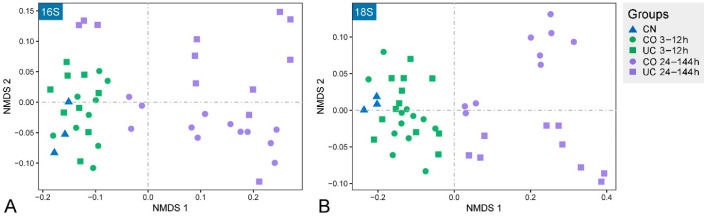
Non-metric multidimensional scaling (NMDS) ordination plots showing the community variations of prokaryotes (**A**) and eukaryotes (**B**). CN: control group, CO: with lid closed, UC: without lid.

Nonmetric multidimensional scaling (NMDS) ordination exhibited a clear aggregation for samples stored for 12 h or less (Figure 4), which was further confirmed by the dissimilarity tests (ANOSIM, PERMANOVA and MRPP) (Table 1). Meanwhile, an obvious segregation of the microbial communities was observed in samples after 24 h storage. As storage time increased, the microbial communities were clearly separated along the first NMDS axis (Figure 4). Mantel test analysis showed that there were significant positive relationships between the Bray–Curtis dissimilarity of microbial communities and storage time in both CO and UC groups (r ≥ 0.789, *p* < 0.001, Figure S3). In addition, compared to the control group, the eukaryotic microbial communities varied significantly after 3 h of storage, whereas the prokaryotic microbial communities did not change significantly within 12 h of storage (Figure 4, Table 1).

The biotic factors that may affect the microbial communities were further investigated by principal component analysis (PCA), which indicated that the microbial communities under different storage conditions were correlated with different taxa (Figure S4). Specifically, the microbial community within 12 h was mainly associated with Actinobacteria, Ciliophora and Bacteroidetes, whereas it correlated with Chloroflexi, Cyanobacteria, Planctomycetes and Verrucomicrobia in samples in sealed containers after storage of 24 h, and Proteobacteria may hold the influential positions in samples without lid over time. The first two principal components accounted for 52.6% of the microbial community variation (34.4% and 18.2% for PC1 and PC2, respectively).

### 3.3. Effect of Sample Storage on Microbial Networks

**Figure 5:**
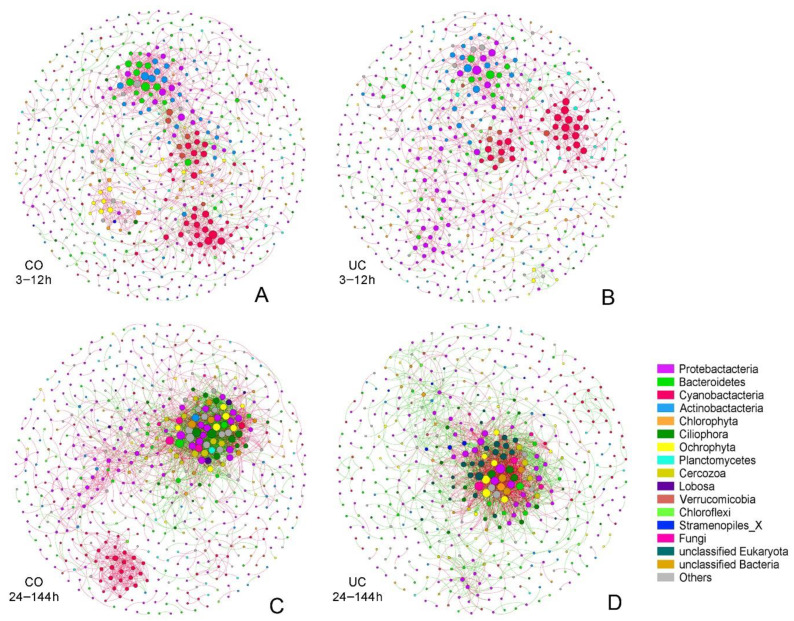
The networks visualizing the OTUs interactions in different groups ((**A**) 3–12 h in CO group; (**B**) 3–12 h in UC group; (**C**) 24–144 h in CO group; (**D**) 24–144 h in UC group). The nodes are colored according to the phylum classification, and the size of each node represents the node degree. Each node corresponds to an OTU, and the edges between nodes correspond to positive (red) or negative (green) correlations. CO: with lid closed, UC: without lid.

To explore the potential influence of storage conditions on microbial interactions, co-occurrence networks were constructed based on OTU relative abundance (Figure 5). During the first 12 h, the network of CO samples had more nodes (667 vs. 639), more edges (1435 vs. 1120) and a higher average connectivity (4.303 vs. 3.505) as compared with the UC group (Table 2). Similarly, the network of the CO group after 24 h also had more nodes (596 vs. 494), more edges (2372 vs. 1883) and were more complex, with the higher average connectivity (7.960 vs. 7.623) and higher average clustering coefficient (0.165 vs. 0.150), compared with the UC network (Table 2). In addition, a higher proportion of positive correlations was observed in samples stored for up to 12 h, whereas negative correlations increased over time (Table 2).

The topological roles of nodes in the network were classified into four categories (i.e., network hubs, module hubs, connectors, and peripherals) based on their within-module connectivity (*Zi*) and among-module connectivity (*Pi*) values (Figure S5). The majority of OTUs were peripherals in both the CO and UC groups. In the early period (3–12 h), the proportion of module hubs was higher in the CO group compared to the UC group (11 vs. 5, Table S1), whereas the latter group had more connectors (6 vs. 1, Table S1), indicating that more interactions between OTUs occurred within their own modules in samples with closed lids, and more connections among taxa occurred in the group without a lid. A similar result was obtained for the later period (24–144 h, Figure S5). The top three keystone taxa (including module hubs and connectors) under different storage conditions are shown in Figure S6. The relative abundance of OTU_794 (Chrysophyceae_Clade-C_X) and OTU_5218 (unclassified_f_Comamonadaceae) was significantly higher in samples stored for 12 h compared to samples stored for 3 or 6 h (*p* < 0.05). After storage for 24 h, the relative abundances of four taxa, i.e., OTU_5218 (unclassified_f_Comamonadaceae), OTU_714 (unclassified_f_Chytridiomycetes), OTU_4441 (unclassified_f_Rhodocyclaceae) and OTU_713 (unclassified_f_Sphaeropleales_X), decreased significantly with storage time (*p* < 0.05), whereas there was a significant increase in the proportions of the other two OTUs, OTU_2770 (*Aphanizomenon*) and OTU_4879 (*Methylotenera*) (*p* < 0.05).

### 3.4. Effect of Sample Storage on the Microbial Community Assembly

**Figure 6:**
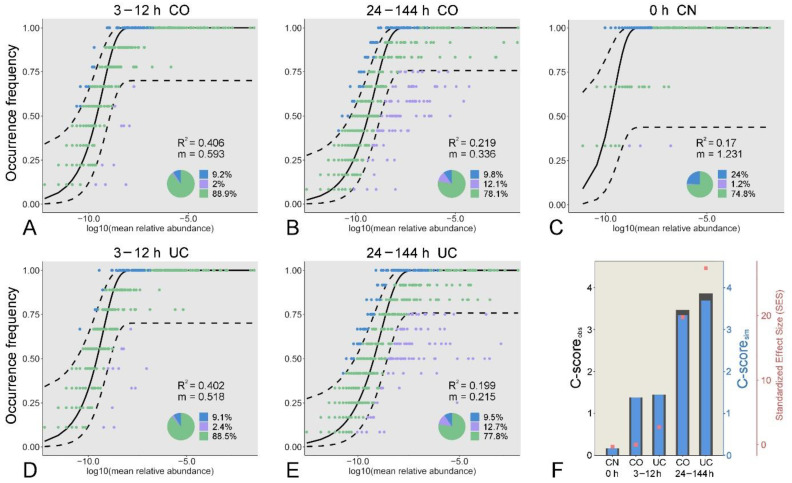
Fit of the occurrence frequency of eukaryotic OTUs as a function of mean relative abundances based on the neutral community model for the microbial communities under different storage conditions (**A**–**E**). The solid black line indicates the best fit to the model, and the dashed black line represents 95% confidence intervals around the model prediction. OTUs that occur within prediction are shown in green, and OTUs that occur more or less frequently than predicted are shown in blue and purple, respectively. R^2^ indicates the fit to the model, and m indicates the immigration rate. C-score metric based on null models (**F**). The values of observed C-score (C-score_obs_) > simulated C-score (C-score_sim_) indicate non-random co-occurrence patterns. Standardized effect size (SES) > 2 and <−2 represent significant segregation and aggregation, respectively. CN: control group, CO: with lid closed, UC: without lid.

The neutral community model (NCM) and checkerboard score (C-score) were used to quantify the effect of sample storage on the microbial community assembly (Figure 6 and Figure S7). The relative contribution of stochastic processes to the prokaryotic and eukaryotic microbial communities increased during the early period (3–12 h), which then decreased over time. Notably, the role of stochastic processes in shaping the eukaryotic microbial community dramatically decreased after storage for 24 h. The proportion of the variation in the occurrence frequency of the eukaryotic microbial communities in the CO group was always slightly higher than that in the UC group, whereas the opposite situation occurred in the prokaryotic microbial communities (Figure 6 and Figure S7). Moreover, the estimated immigration rate decreased gradually with storage time, and it was higher for the CO communities than for the UC samples, indicating that the dispersal ability was probably more active in the closed-lid samples. In addition, the checkerboard score (C-score) results showed that the observed C-score values (C-score_obs_) were slightly higher than the simulated values (C-score_sim_), regardless of the storage conditions (Figure 6 and Figure S7, Table S2). Overall, the standardized effect size (SES) increased with storage time, suggesting that deterministic processes were becoming more important in shaping the microbial communities over time.

## 4. Discussion

Keeping samples frozen or using a preservative are considered the best alternative solutions for sample storage during shipping, although it has been shown that room-temperature storage for a few days would not strongly affect the overall bacterial community structure of soil or fecal samples in some cases [17,18]. However, it is quite challenging to store large-volume-of-water samples at low temperature during remote fieldwork. To obtain a comprehensive understanding of how storage conditions impact the whole microbial community of water samples, we revealed the detailed changes of both prokaryotic and eukaryotic microbial communities under different storage conditions over time. We further explored the potential microbial interactions and the ecological processes in driving the microbial communities during storage.

The alpha diversity of both prokaryotes and eukaryotes are not expected to increase over storage time, as some inactive or dead species may not be detected in the later period of sample storage [13]. Surprisingly, however, the prokaryotic diversity in the samples with lid closed increased significantly after 24 h storage compared to the control group (Figure 3). In contrast to previous reports [12], the eukaryotic richness in the 24–144 h group was also remarkably higher than in the initial community, irrespective of lid status. The micro-environmental change during storage may benefit the growth of some dormant/rare species and contribute to the detection of higher diversity in the samples after 24 h storage, indicating that the microbial diversity changed rapidly under different environmental conditions [22,54,55]. The NMDS analysis showed that the microbial communities were affected by both closed lid and storage time, especially when samples were stored for more than 12 h (Figure 4). The dissimilarity tests further suggested that samples should be processed immediately (within 3 h) if the eukaryotic microorganisms are the target taxa, whereas a dramatic change of the prokaryotic microbial community was found after 12 h storage (Table 1). This is consistent with the fact that eukaryotes are more sensitive to environmental changes [56,57].

Uneven or non-specific PCR amplification efficiencies and the differential gene copy number per cell/individual are known to be inherent biases of PCR-based sequencing technology [58,59]. Thus, the observed changes in relative abundances do not necessarily reflect a true change in the absolute abundance. In this study, triplicate PCRs were performed to minimize the PCR bias effects, which are supposed to apply equally to all samples within a specific environment [60,61]. With the increase of storage time, it was presumed that sealed bottles created a hypoxic environment, which would be unsuitable for aerobic microorganisms to thrive [62,63]. After 12 h of storage, the relative abundance of aerobic Proteobacteria (*Pseudomonas* and unclassified Comamonadaceae) were dramatically lower in the low-oxygen habitat (CO) than in the UC group (Figure 2D). By contrast, a micro-aerobic environment was maintained for the samples stored without lid by allowing gas exchange across the air–water interface, which may inhibit the growth and propagation of anaerobic microorganisms [64]. The proportions of *Synechococcus*, hgcI_clade, CL500-29_marine_group, LD12 and MWH-UniP1_aquatic_group were significantly higher under the low-oxygen conditions in the CO group than in the UC group (Figure 2D), which is consistent with previous results that anaerobic microorganisms were negatively correlated with the oxygen level [65,66]. Interestingly, a member of aerobic or facultative anaerobic bacteria, Planctomycetes (Phycisphaeraceae and Planctomycetaceae) [67], was observed with higher abundance under conditions of hypoxia (CO), supporting the idea that aerobic bacteria may still function under anoxic conditions [68].

The effects of nutrient variation on microorganisms under different storage conditions should also be taken into account. The dramatic decrease of *Chlamydomonas*, hgcI_clade, CL500-29_marine_group, norank_f__LD12_freshwater_group and *Synechococcus* over storage time could be partially attributed to nitrogen or phosphorus consumption and deficiency, as previously shown [66,69]. This could further influence the ciliate community as the proportion of their potential prey (i.e., small phytoplankton and bacteria) may decrease over time. Previous studies showed that the tintinnids community can be strongly affected by the resource availability [70], which may explain the significant decrease in this group after 12 h of storage. Conversely, as observed in this study, the lack of nutrients may benefit the growth of Ochrophyta, which are frequently dominant in oligotrophic lakes [71,72].

This study highlighted the effect of possible interactions between microorganisms on microbial community patterns. Co-occurrence network analysis revealed more frequent interactions between Cyanobacteria and other microorganisms, which may be ascribed to the influence of the dissolved organic matter from cyanobacterial debris on microbial community structure [73]. Compared to the later period of storage, co-occurrence positive correlations between Cyanobacteria taxa were much stronger during the first 12 h (Figure 5). One explanation is that the consumption of nutrients with the growth of Cyanobacteria resulted in nutrient deficiency in the later period, which in turn increased inter-phylum competition between Cyanobacterial taxa [74]. It has been reported that once the environment was suitable, the number of Cyanobacteria would dramatically increase, as most Cyanobacteria taxa share a similar niche [75]. Meanwhile, dormant microorganisms may revive following environmental change, which could produce more complex interactions in the network [54]. Moreover, the conspicuous co-occurrence pattern between aerobic microorganisms (e.g., Actinobacteria, Bacteroidetes and Proteobacteria nodes in Figure 5A,B) suggests that the aerobic microorganisms had strong positive correlations in the early period of storage. A remarkable positive correlation between two photosynthetic phyla, Chlorophyta and Ochrophyta, was also observed during the first 12 h storage in the group without adequate light (CO), which reminds us that the light transparency of the sampling bottles is also a major consideration for sample storage. In addition, the decrease of oxygen levels over time may change the interspecific interactions and the co-occurrence pattern between aerobic microorganisms (e.g., more Ciliophora and less Bacteroidetes, Actinobacteria nodes in Figure 5C,D).

Although the contribution of the ecological processes to microbial community assembly varied slightly under different storage conditions, the relative contribution of deterministic processes played an increasingly important role in the assembly of microbial communities (Figure 6 and Figure S7). The continuously decreasing concentration of dissolved oxygen in the sample caused by microbial activity and respiration may impose a strong selective environmental filter on the community structure [19,20,63]. On the other hand, microbial interactions contributed to deterministic community assembly [76], as indicated by the complex co-occurrence networks, where the percentage of negative edges gradually increased over time. Moreover, the value of the standardized effect size (SES) increased notably with the storage time, indicating that the roles of deterministic processes become increasingly important in the community assembly, especially in the eukaryotic microbial community [47].

## 5. Conclusions

This study underlined the importance of the effects of sample storage conditions on both prokaryotic and eukaryotic microorganisms. Our results indicated that the eukaryotic microbial community of freshwater samples changed at a faster rate than the bacterial community during room-temperature storage, regardless of the storage conditions. The eukaryotic beta diversity changed significantly between the control and treatment samples stored for more than 3 h, whereas the bacterial community was largely unaffected by container status or storage time within 12 h of storage at room temperature. The potential interactions between microbial taxa were more complex when samples were stored in bottles with lids. The deterministic processes played an increasingly important role in shaping the freshwater microbial communities with storage time. These results suggest that water samples collected and stored without refrigeration for no more than 12 h may still be useful for bacterial community analyses. If the eukaryotic microbial communities are desired, storage of water samples should be limited to 3 h at room temperature.

## Figures and Tables

**Table 1:** Dissimilarity tests showing the differences of microbial communities under different storage conditions based on the Bray–Curtis distances.

Groups			ANOSIM		PERMANOVA		MRPP	
			R	*p*	R^2^	*p*	Delta	*p*
Prokaryotes	3–12 h	CO vs. UC	−0.085	0.900	0.024	0.861	0.316	0.924
		CO vs. CN	−0.066	0.578	0.118	0.216	0.302	0.097
		UC vs. CN	−0.126	0.746	0.103	0.324	0.315	0.155
	24–144 h	CO vs. UC	0.571	**0.001**	0.245	**0.001**	0.504	**0.001**
		CO vs. CN	0.670	**0.004**	0.320	**0.004**	0.447	**0.005**
		UC vs. CN	0.883	**0.001**	0.347	**0.002**	0.535	**0.002**
Eukaryotes	3–12 h	CO vs. UC	0.048	0.245	0.066	0.330	0.302	0.256
		CO vs. CN	0.464	**0.011**	0.276	**0.009**	0.311	**0.005**
		UC vs. CN	0.479	**0.006**	0.281	**0.006**	0.325	**0.006**
	24–144 h	CO vs. UC	0.175	**0.030**	0.123	**0.024**	0.540	**0.014**
		CO vs. CN	0.433	**0.013**	0.299	**0.008**	0.510	**0.001**
		UC vs. CN	0.367	**0.038**	0.265	**0.003**	0.571	**0.005**

ANOSIM: analysis of similarities; PERMANOVA: permutational multivariate analysis of variance; MRPP: multiple-response permutation procedure. Significant differences (*p* < 0.05) are shown in bold. CN: control group, CO: with lid closed, UC: without lid.

**Table 2:** Network topological properties of CO and UC networks.

Network Topological Properties	3–12 h		24–144 h	
	CO	UC	CO	UC
Nodes	667	639	596	494
Edges	1435	1120	2372	1883
R^2^ of power law	0.918	0.905	0.780	0.799
Average clustering coefficient (avgCC)	0.204	0.204	0.165	0.150
Average connectivity (avgK)	4.303	3.505	7.960	7.623
Average geodesic distance (GD)	7.616	7.100	5.625	4.960
Geodesic efficiency I	0.185	0.181	0.237	0.264
Positive edges	0.815	0.828	0.536	0.492

CO: with lid closed, UC: without lid.

## Data Availability

The datasets presented in this study can be found in online repositories. The names of the repository/repositories and accession number(s) can be found from the NCBI database: PRJNA777784.

## Supplementary Materials

The following supporting information can be downloaded at: https://www.mdpi.com/article/10.3390/microorganisms10061176/s1, Figure S1. Stacked bar charts of the relative abundance of the prokaryotes (A) and eukaryotes (B) at the phylum level. Shown are the averages from *n* = 3. CN: control group, CO: with lid closed, UC: without lid. Figure S2. Significant differences analysis for eukaryotes between CO (with lid closed) and UC (without lid) at phylum level (A,C) and genus level (B,D) (the top 15). Shown are the averages from *n* = 3. *: 0.01 < *p* ≤ 0.05, **: 0.001 < *p* ≤ 0.01, ***: *p* ≤ 0.001. Figure S3. Mantel tests between Bray-Curtis dissimilarity of prokaryotic (A,B) and eukaryotic (C,D) microbial communities and storage time. The solid black line indicates the best fit, and the grey shaded area around the lines represents 95% confidence intervals. *p* value and r refer to Mantel tests of Spearman’s rank correlation. CO: with lid closed, UC: without lid. Figure S4. Principal component analysis (PCA) biplots for microbial community samples. Arrows indicate important taxa (top 15) with regard to sample clusters at the phylum level. CN: control group, CO: with lid closed, UC: without lid. Figure S5. The network nodes were classified by within-module connectivity (*Zi*) and among-module connectivity (*Pi*), with the threshold values of 2.5 and 0.62 to categorize OTUs. CO: with lid closed, UC: without lid. Figure S6. The relative abundance of potential keystone taxa (top 3) under different storage conditions (A: CO, 3–12 h, B: UC, 3–12 h, C: CO, 24–144 h, D: UC, 24–144 h). Data are presented as mean ± SD. Different letters indicate a statistical difference (*p* < 0.05, ANOVA) between groups. CO: with lid closed, UC: without lid, E: Eukaryotes, P: Prokaryotes. 1: photoheterotrophs, 2: aerobes or facultative anaerobes, 3: aerobes, 4: unknown, 5: anaerobes, 6: photoheterotrophs, aerobes, anaerobes or facultative anaerobes. Figure S7. Fit of the occurrence frequency of prokaryotic OTUs as a function of mean relative abundances based on the neutral community model for the microbial communities under different storage conditions (A–E). The solid black line indicates the best fit to the model, and the dashed black line represents 95% confidence intervals around the model prediction. OTUs that occur within prediction are shown in green, and OTUs that occur more or less frequently than predicted are shown in blue and purple, respectively. R^2^ indicates the fit to the model, and m indicates the immigration rate. C-score metric based on null models (F). The values of observed C-score (C-score_obs_) > simulated C-score (C-score_sim_) indicate non-random co-occurrence patterns. Standardized effect size (SES) > 2 and <−2 represent significant segregation and aggregation, respectively. CN: control group, CO: with lid closed, UC: without lid. Table S1. The topological characteristics of nodes (OTUs) in the networks. Table S2. Observed C-scores, simulated C-scores, and standardized effect sizes for microbial communities under different storage conditions.
