# Supplementary material for: Changes of the Freshwater Microbial Community Structure and Assembly Processes during Different Sample Storage Conditions
Source: Microorganisms. 2022 Jun 8;10(6):1176. doi: 10.3390/microorganisms10061176 (PMC9229623; doi:10.3390/microorganisms10061176)
Supplement: Supplementary file 1 [file microorganisms-10-01176-s001.zip › microorganisms-1749192-supplementary.pdf]

Supplementary Materials

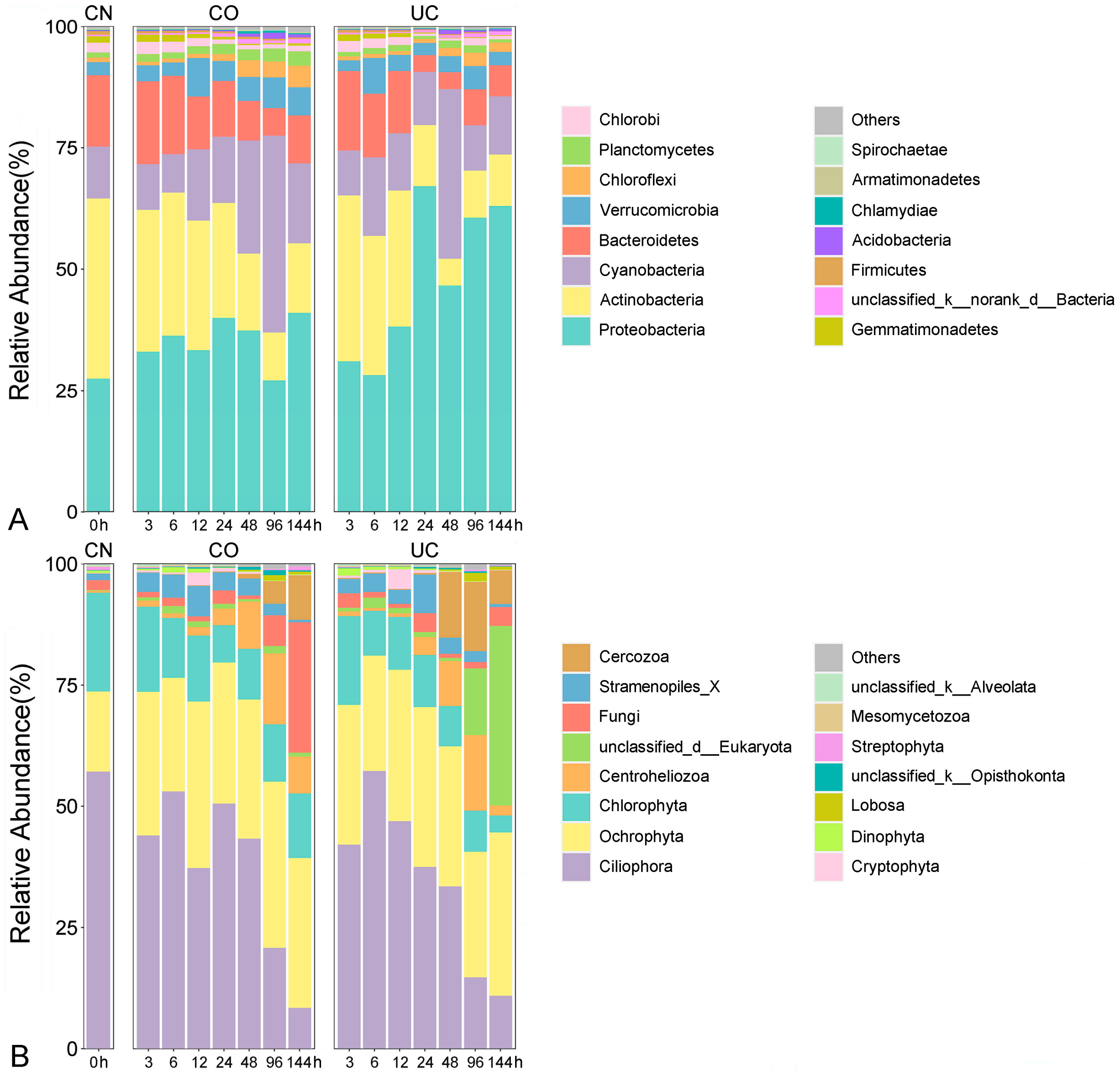

Figure S1. Stacked bar charts of the relative abundance of the prokaryotes (A) and eukaryotes (B) at the phylum level. Shown are the averages from n = 3. CN: control group, CO: with lid closed, UC: without lid.

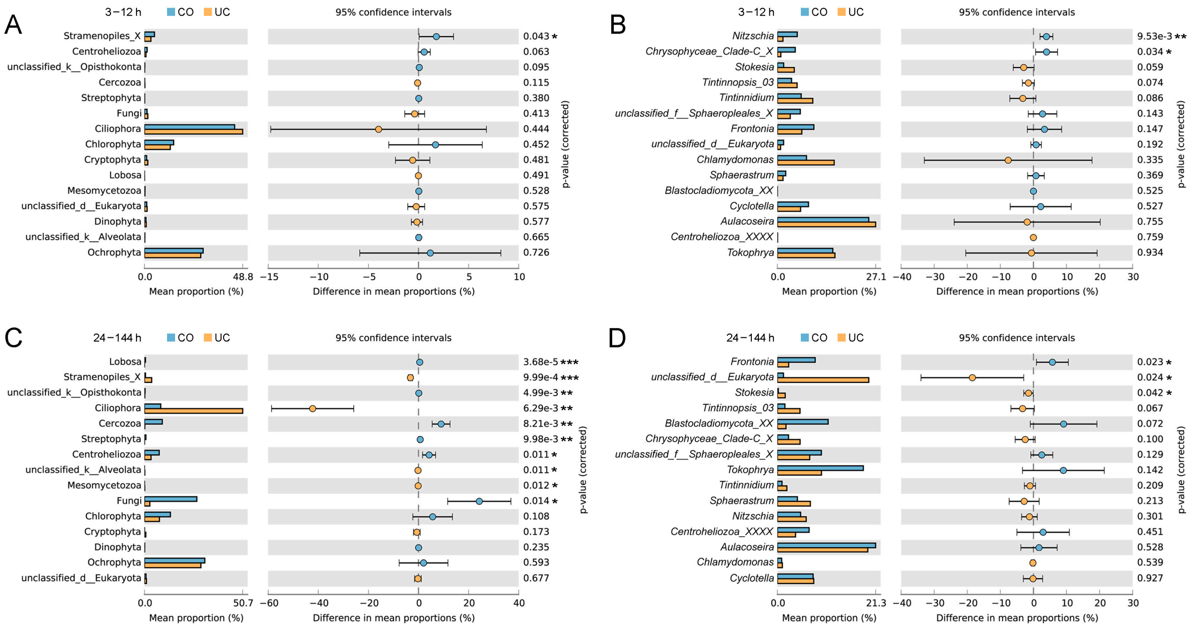

Figure S2. Significant differences analysis for eukaryotes between CO (with lid closed) and UC (without lid) at phylum level (A, C) and genus level (B, D) (the top 15). Shown are the averages from n = 3. *: $0.01 < P \leq 0.05$, **: $0.001 < P \leq 0.01$, ***: $P \leq 0.001$.

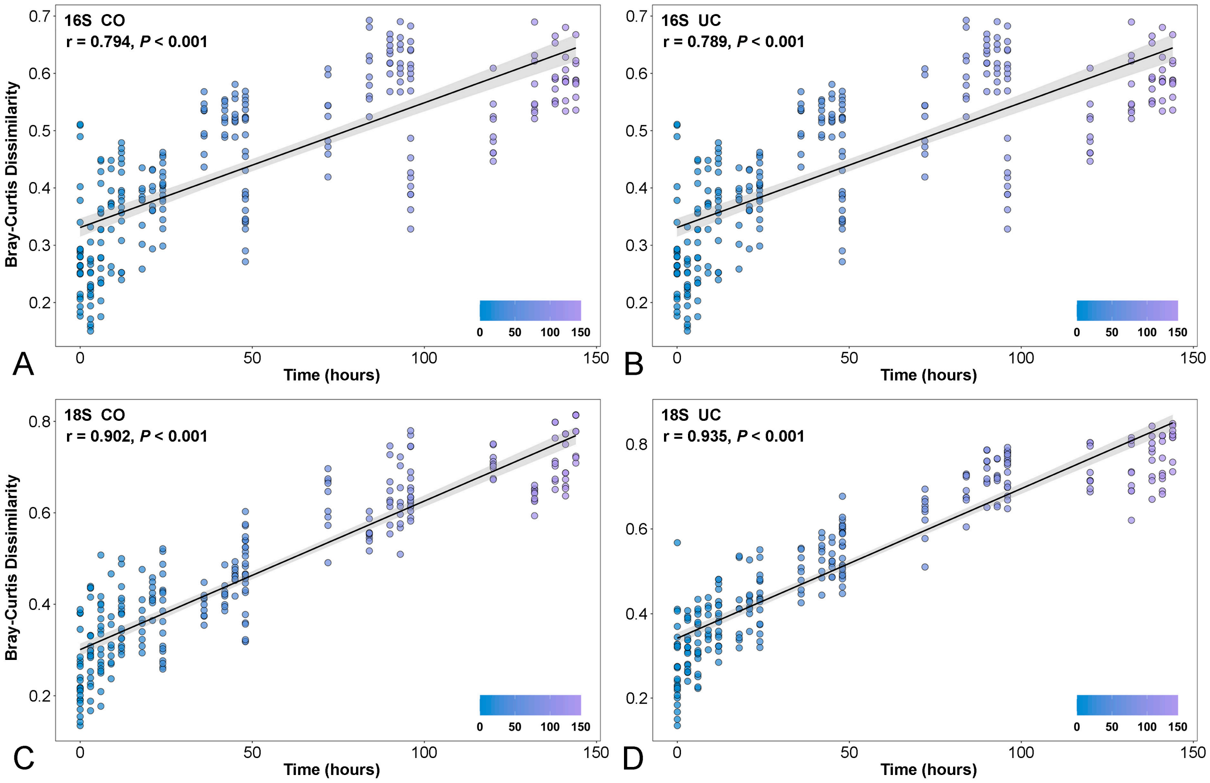

Figure S3. Mantel tests between Bray-Curtis dissimilarity of prokaryotic (A, B) and eukaryotic (C, D) microbial communities and storage time. The solid black line indicates the best fit, and the grey shaded area around the lines represents 95% confidence intervals. *P* value and r refer to Mantel tests of Spearman's rank correlation. CO: with lid closed, UC: without lid.

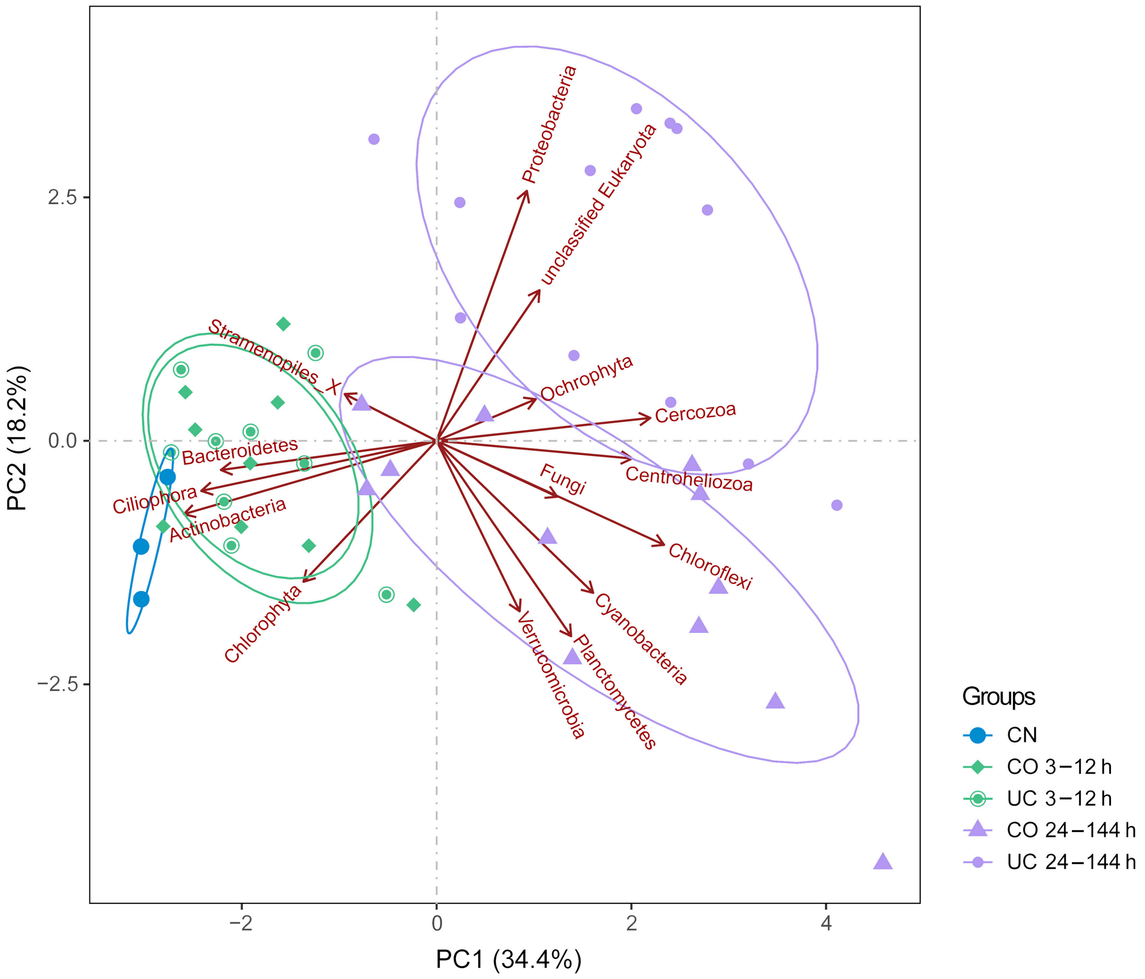

Figure S4. Principal component analysis (PCA) biplots for microbial community samples. Arrows indicate important taxa (top 15) with regard to sample clusters at the phylum level. CN: control group, CO: with lid closed, UC: without lid.

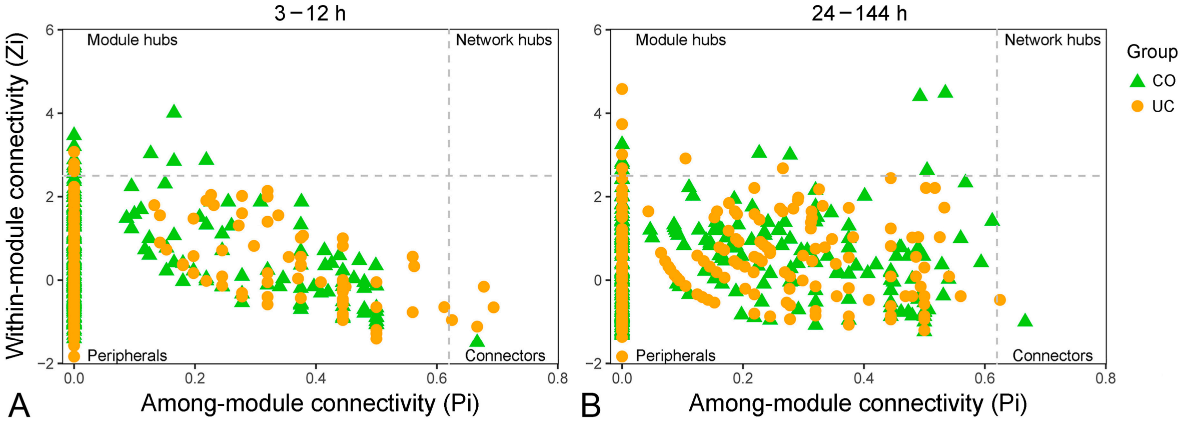

Figure S5. The network nodes were classified by within-module connectivity (*Zi*) and among-module connectivity (*Pi*), with the threshold values of 2.5 and 0.62 to categorize OTUs. CO: with lid closed, UC: without lid.

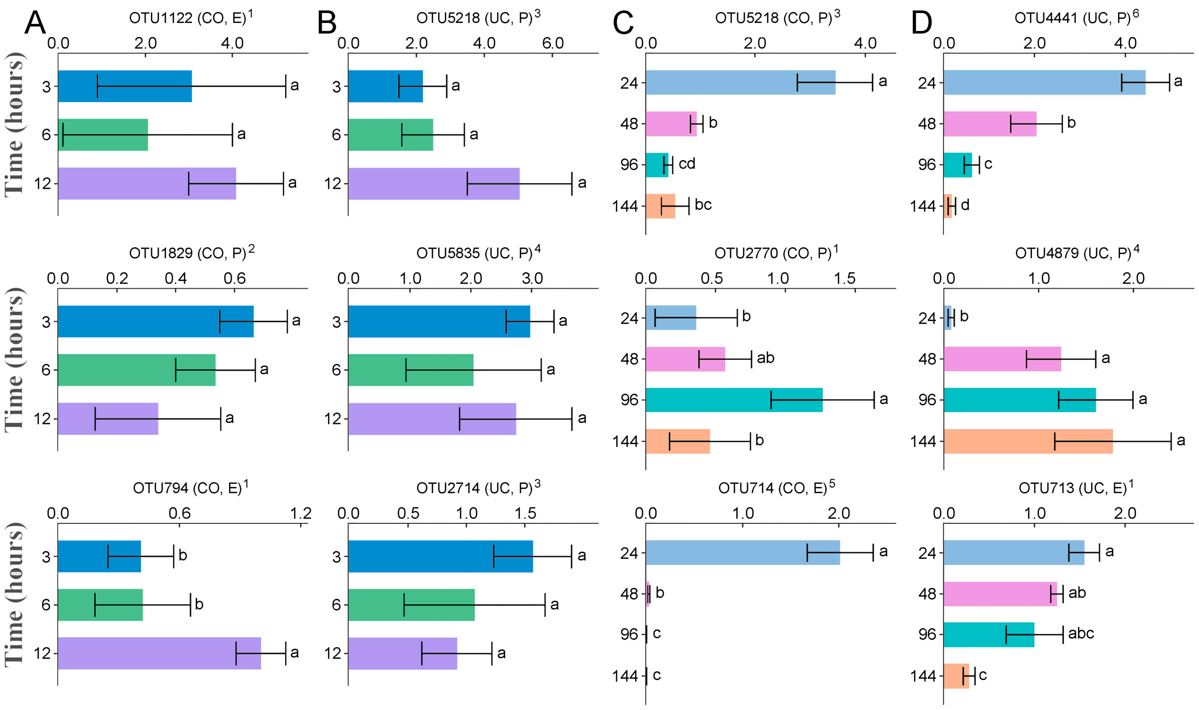

Figure S6. The relative abundance of potential keystone taxa (top 3) under different storage conditions (A: CO, 3-12 h, B: UC, 3-12 h, C: CO, 24-144 h, D: UC, 24-144 h). Data are presented as mean ± SD. Different letters indicate a statistical difference ($P < 0.05$, ANOVA) between groups. CO: with lid closed, UC: without lid, E: Eukaryotes, P: Prokaryotes. 1: photoheterotrophs, 2: aerobes or facultative anaerobes, 3: aerobes, 4: unknown, 5: anaerobes, 6: photoheterotrophs, aerobes, anaerobes or facultative anaerobes.

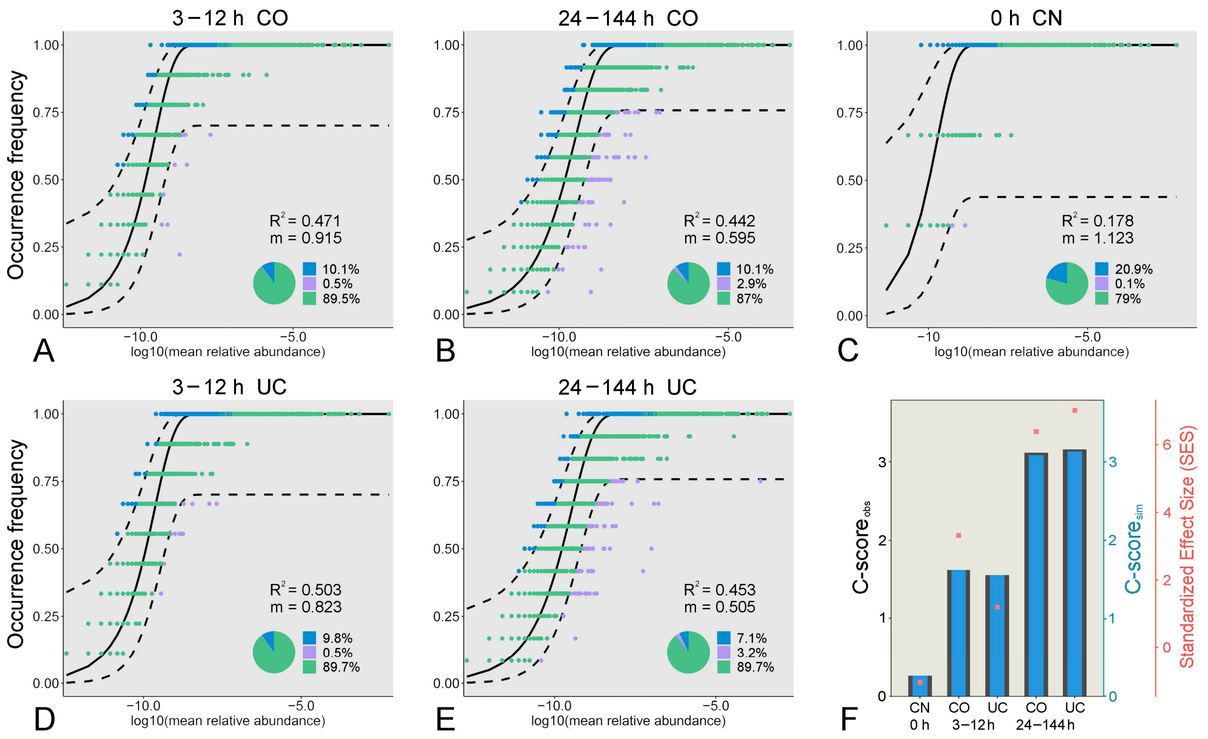

Figure S7. Fit of the occurrence frequency of prokaryotic OTUs as a function of mean relative abundances based on the neutral community model for the microbial communities under different storage conditions (A-E). The solid black line indicates the best fit to the model, and the dashed black line represents 95% confidence intervals around the model prediction. OTUs that occur within prediction are shown in green, and OTUs that occur more or less frequently than predicted are shown in blue and purple, respectively. R^2 indicates the fit to the model, and m indicates the immigration rate. C-score metric based on null models (F). The values of observed C-score (C-score$_{obs}$) > simulated C-score (C-score$_{sim}$) indicate non-random co-occurrence patterns. Standardized effect size (SES) > 2 and < -2 represent significant segregation and aggregation, respectively. CN: control group, CO: with lid closed, UC: without lid.

Supplementary Tables

Table S1. The topological characteristics of nodes (OTUs) in the networks.

OTU ID	Category	Time	Storage	Taxonomy (Domain; Kingdom; Phylum; Class; Order; Family; Genus)
OTU_3249	connectors	3-12 h	CO	Bacteria; norank Bacteria; Proteobacteria; Gammaproteobacteria; Legionellales; Coxiellaceae; *Coxiella*
OTU_1975	connectors	3-12 h	UC	Bacteria; norank Bacteria; Proteobacteria; Betaproteobacteria; Rhodocyclales; Rhodocyclaceae; unclassified Rhodocyclaceae
OTU_2616	connectors	3-12 h	UC	Bacteria; norank Bacteria; Cyanobacteria; Cyanobacteria; SubsectionI; FamilyI SubsectionI; unclassified FamilyI SubsectionI
OTU_2876	connectors	3-12 h	UC	Bacteria; norank Bacteria; Planctomycetes; Planctomycetacia; Planctomycetales; Planctomycetaceae; *Pirellula*
OTU_2979	connectors	3-12 h	UC	Bacteria; norank Bacteria; Planctomycetes; Planctomycetacia; Planctomycetales; Planctomycetaceae; norank Planctomycetaceae
OTU_5755	connectors	3-12 h	UC	Bacteria; norank Bacteria; Actinobacteria; Actinobacteria; PeM15; norank PeM15; norank PeM15
OTU_5835	connectors	3-12 h	UC	Bacteria; norank Bacteria; Actinobacteria; Actinobacteria; Acidimicrobiales; Acidimicrobiaceae; CL500-29 marine group
OTU_309	connectors	24-144 h	CO	Eukaryota; Alveolata; Ciliophora; Phyllopharyngea; Cyrtophoria; Chilodonellidae; Chilodonellidae X
OTU_1087	connectors	24-144 h	UC	Eukaryota; unclassified Eukaryota; unclassified Eukaryota; unclassified Eukaryota; unclassified Eukaryota; unclassified Eukaryota; unclassified Eukaryota
OTU_370	module hubs	3-12 h	CO	Bacteria; norank Bacteria; Firmicutes; Bacilli; Bacillales; unclassified Bacillales; unclassified Bacillales
OTU_720	module hubs	3-12 h	CO	Eukaryota; Alveolata; Ciliophora; Spirotrichea; Hypotrichia; Halteriidae; unclassified Halteriidae
OTU_794	module hubs	3-12 h	CO	Eukaryota; Stramenopiles; Ochrophyta; Chrysophyceae; Chrysophyceae X; Chrysophyceae Clade-C; Chrysophyceae Clade-C X
OTU_1122	module hubs	3-12 h	CO	Eukaryota; Stramenopiles; Ochrophyta; Bacillariophyta; Bacillariophyta X; Polar centric Mediophyceae; *Cyclotella*
OTU_1829	module hubs	3-12 h	CO	Bacteria; norank Bacteria; Bacteroidetes; Sphingobacteriia; Sphingobacteriales; Chitinophagaceae; unclassified Chitinophagaceae
OTU_2006	module hubs	3-12 h	CO	Bacteria; norank Bacteria; Cyanobacteria; Cyanobacteria; SubsectionI; FamilyI SubsectionI; *Microcystis*
OTU_2011	module hubs	3-12 h	CO	Bacteria; norank Bacteria; Bacteroidetes; Flavobacteriia; Flavobacteriales; unclassified Flavobacteriales; unclassified Flavobacteriales
OTU_2063	module hubs	3-12 h	CO	Bacteria; norank Bacteria; Bacteroidetes; Sphingobacteriia; Sphingobacteriales; ST-12K33; norank ST-12K33
OTU_2712	module hubs	3-12 h	CO	Bacteria; norank Bacteria; Proteobacteria; Betaproteobacteria; Betaproteobacteria Incertae Sedis; Unknown Family Betaproteobacteria Incertae Sedis; *Chitinivorax*
OTU_2770	module hubs	3-12 h	CO	Bacteria; norank Bacteria; Cyanobacteria; Cyanobacteria; SubsectionIV; FamilyI SubsectionIV; *Aphanizomenon*
OTU_5774	module hubs	3-12 h	CO	Bacteria; norank Bacteria; Cyanobacteria; Cyanobacteria; norank Cyanobacteria; norank Cyanobacteria; norank Cyanobacteria

OTU_917	module hubs	3-12 h	UC	Eukaryota; Alveolata; unclassified Alveolata; unclassified Alveolata; unclassified Alveolata; unclassified Alveolata; unclassified Alveolata
OTU_2004	module hubs	3-12 h	UC	Bacteria; norank Bacteria; Bacteroidetes; Sphingobacteriia; Sphingobacteriales; NS11-12 marine group; norank NS11-12 marine group
OTU_2512	module hubs	3-12 h	UC	Bacteria; norank Bacteria; Proteobacteria; Alphaproteobacteria; Rhizobiales; Methylocystaceae; unclassified Methylocystaceae
OTU_2714	module hubs	3-12 h	UC	Bacteria; norank Bacteria; Bacteroidetes; Sphingobacteriia; Sphingobacteriales; Chitinophagaceae; *Terrimonas*
OTU_5218	module hubs	3-12 h	UC	Bacteria; norank Bacteria; Proteobacteria; Betaproteobacteria; Burkholderiales; Comamonadaceae; unclassified Comamonadaceae
OTU_714	module hubs	24-144 h	CO	Eukaryota; Opisthokonta; Fungi; Chytridiomycota; Chytridiomycotina; Chytridiomycetes; unclassified Chytridiomycetes
OTU_1105	module hubs	24-144 h	CO	Bacteria; norank Bacteria; Bacteroidetes; Cytophagia; Cytophagales; Cyclobacteriaceae; norank Cyclobacteriaceae
OTU_1164	module hubs	24-144 h	CO	Eukaryota; Stramenopiles; Ochrophyta; Synurophyceae; Synurales; Synurales X; *Mallomonas*
OTU_2686	module hubs	24-144 h	CO	Bacteria; norank Bacteria; Proteobacteria; Betaproteobacteria; Rhodocyclales; Rhodocyclaceae; 12up
OTU_2770	module hubs	24-144 h	CO	Bacteria; norank Bacteria; Cyanobacteria; Cyanobacteria; SubsectionIV; FamilyI SubsectionIV; *Aphanizomenon*
OTU_3368	module hubs	24-144 h	CO	Bacteria; norank Bacteria; Proteobacteria; Alphaproteobacteria; Rhodospirillales; Acetobacteraceae; *Roseomonas*
OTU_5179	module hubs	24-144 h	CO	Bacteria; norank Bacteria; Chlorobi; Chlorobia; Chlorobiales; OPB56; norank OPB56
OTU_5218	module hubs	24-144 h	CO	Bacteria; norank Bacteria; Proteobacteria; Betaproteobacteria; Burkholderiales; Comamonadaceae; unclassified Comamonadaceae
OTU_5640	module hubs	24-144 h	CO	Bacteria; norank Bacteria; Bacteroidetes; Flavobacteriia; Flavobacteriales; Cryomorphaceae; *Fluviicola*
OTU_713	module hubs	24-144 h	UC	Eukaryota; Archaeplastida; Chlorophyta; Chlorophyceae; Sphaeropleales; Sphaeropleales X; unclassified Sphaeropleales X
OTU_2742	module hubs	24-144 h	UC	Bacteria; norank Bacteria; Actinobacteria; Actinobacteria; Corynebacteriales; Mycobacteriaceae; *Mycobacterium*
OTU_4441	module hubs	24-144 h	UC	Bacteria; norank Bacteria; Proteobacteria; Betaproteobacteria; Rhodocyclales; Rhodocyclaceae; unclassified Rhodocyclaceae
OTU_4462	module hubs	24-144 h	UC	Bacteria; norank Bacteria; Proteobacteria; Gammaproteobacteria; Pseudomonadales; Pseudomonadaceae; *Pseudomonas*
OTU_4879	module hubs	24-144 h	UC	Bacteria; norank Bacteria; Proteobacteria; Betaproteobacteria; Methylophilales; Methylophilaceae; *Methylotenera*
OTU_5475	module hubs	24-144 h	UC	Bacteria; norank Bacteria; Proteobacteria; Betaproteobacteria; Burkholderiales; Comamonadaceae; unclassified Comamonadaceae
OTU_5639	module hubs	24-144 h	UC	Bacteria; norank Bacteria; Proteobacteria; Betaproteobacteria; Rhodocyclales; Rhodocyclaceae; *Dechloromonas*

CO: with lid closed, UC: without lid.

Table S2. Observed C-scores, simulated C-scores, and standardized effect sizes for microbial communities under different storage conditions.

**Microorganism**	**Groups**	**C-score$_{obs}$**	**C-score$_{sim}$**	**SES**
Prokaryotes	CN 0 h	0.26278	0.26291	-1.0642
	CO 3-12 h	1.6215	1.6194	3.2724
	UC 3-12 h	1.5557	1.5547	1.1672
	CO 24-144 h	3.1194	3.0889	6.3187
	UC 24-144 h	3.1585	3.132	6.9232
Eukaryotes	CN 0 h	0.16824	0.16838	-0.30359
	CO 3-12 h	1.3873	1.3873	-0.013559
	UC 3-12 h	1.4485	1.4442	2.6704
	CO 24-144 h	3.491	3.3616	19.671
	UC 24-144 h	3.8576	3.6856	27.053

C-score significantly larger than expected by chance and high standardized effect size (SES) value (> 2) suggest significant separation, C-score that is markedly smaller than expected by chance and low SES value (< -2) indicate a significant aggregated pattern. Obs, observed index, Sim, simulated index, CN: control group, CO: with lid closed, UC: without lid.
